# The New York Homeopathic Union

**Published:** 1897-07

**Authors:** Emma D. Wilcox

**Affiliations:** Secretary


### THE NEW YORK HOMEOPATHIC UNION.

The regular monthly meeting of the New York Homeopathic Union was held at 62 West Forty-ninth Street, New York, Thursday evening, June 17th, 1897, the President, Edmund Carleton, M. D., in the chair.

Members present: Drs. Clark, Clock, Finch, Wilcox, and Young. Dr. Stanton was obliged to go early, and left his paper, on "Dysuria," to be read by the Secretary. (This paper will be found at the end of this report.)

Reading and comment upon the "Defense of *The Organon*" was continued to the foot of the seventy-ninth page. The hour of 9 P. M. having arrived, the defense was then laid aside, and the meeting became clinical.

Dr. Wilcox related the history of a case of urinary calculus, which had come under her personal observation. The patient was a man of full habit, who had passed through the Morphine experience in four previous attacks, usually requiring about twelve hours each to stop the pain. His wife appeared at the Doctor's office in the night, begging for the immediate injection of Morphine, so terrible was the suffering. It was only by promising relief in half an hour that reluctant assent could be gained from man and wife to the employment of Homeopathy instead of allopathy. Flushed face; screaming and tossing; cramping pains in region of left kidney, coming in paroxysms of terrible severity. These were sudden in coming and going. Continuous hard pain between the paroxysms. Belladonna cm (Fincke), was dissolved in water and given in repeated doses—the second five minutes after the first, and the third ten minutes after the second. Dosed for about ten minutes and awoke in terrible paroxysms of pain, which lasted seven minutes. Another dose was given. Before the hour was up patient went to sleep, and two hours after was reported still sleeping. Awoke free from pain. Medicine discontinued. Next day he went down-town to business, forgetting, by the way, to save the urine for inspection. Undoubtedly the customary exodus of gravel took place. It was a rainy day, and he got wet. The following midnight he awoke with a dull pain, which lasted less than an hour and has not returned. Owing to indigestion and constipation three days later he received a dose of Nux-vomica. There was no further complaint.

Dr. Young had had a similar case, cured with Belladonna. He was fortunate enough to get the specimen of gravel that passed, which was angular and sharp, about the size of a squirrel shot. He believed that Belladonna was the remedy generally indicated in these cases.

Dr. Finch agreed to this proposition, and added that the same remedy was frequently of service in pains produced by sounding. The long, red radish is said to have dissolved stone.

Dr. Wilcox asked if anything could be done between attacks to head them off.

Dr. Carleton said that all symptoms existing between the attacks should be sought with diligence and studied carefully, as they are as valuable in cases of calculus as the symptoms of the apyrexia are in cases of intermittent fever. Hahnemann has shown how important they are, and all of us, no doubt, have proved by experience the truth of his statement.

Dr. Young observed a case that had been under the care of many physicians. They all had treated her for calculus. She had taken much Opium, Calomel, and Quinine, besides the waters of Carlsbad, and all that sort of thing. After a while jaundice developed, and a sore spot over the gall bladder. She was yellow a long time. Patient was fretful and easily made to cry. The attacks came on at night, with sore throat, sore and sensitive stomach and gall-bladder. Radiating, shooting pains, worse when lying, better from motion; loose, green stools, worse at night. Pulsatilla cured.

Dr. Clark said that he prescribed for one case of calculus to the best of his ability, upon indications, for eighteen hours, but unsuccessfully. Then he gave Morphine until the stone passed. It was of the uric acid variety. No uric acid had been found in the urine. The next case was relieved with Berberis. Stone not found. The third case was brought on by drinking cider. Patient was doubled up with colic, which was somewhat relieved by hard pressure. Colocynth relieved. Sharp stone, about the size of the head of a pin. His fourth case was complicated with heart and intestinal troubles, and had been the subject of a number of previous attacks of stone. All the symptoms led him to give Nux-vomica, which relieved. No stone was recovered. He has never succeeded in getting so speedy relief as he would like, with the indicated remedy alone. He considers the waters of Contrexeville, France, the best for dissolving uric acid calculus (only). Between attacks he studied Berb., Lyc., Osmium, Sepia. He called attention to a kidney that had been extirpated, containing eighteen uric acid stones. No previous symptoms of uric acid had been seen.

Dr. Clock reported the case of a boy of twelve, who used to wet the bed at night and then awake crying with pain. Consulting Lilienthal for nocturnal enuresis, she found Equisetum to have the pain symptom described. The materia medica showed a correspondence with all symptoms of the boy with those of Equisetum. The two hundredth cured. A man over fifty years of age had frequent attacks of renal colic. These were treated allopathically with Morphine, each succeeding time with larger doses. He had great dread of the attacks, and decided to try Homeopathy. A red-faced man, of full habit, heavy drinker and high liver, had taken many strong drugs, and said that no small doses would help him. Pains dreadful; yelling and tossing about; sand in urine; pain along ureters, while sand and urine passed. Nux-vomica, two hundredth, cured.

It being 10 o'clock the remaining papers, with discussion thereon, were postponed to the next meeting.

Adjourned to the third Thursday in October, the next regular meeting, as the Union does not meet in July, August, and September.

EMMA D. WILCOX,
*Secretary.*

